# Differentiation during fig ontogeny suggests opposing selection by mutualists

**DOI:** 10.1002/ece3.5918

**Published:** 2020-01-07

**Authors:** Silvia B. Lomáscolo, Douglas J. Levey

**Affiliations:** ^1^ Instituto de Ecología Regional CONICET‐Universidad Nacional de Tucumán Residencia Universitaria Horco Molle Yerba Buena Argentina; ^2^ Population and Community Ecology Division of Environmental Biology National Science Foundation Alexandria Virginia

**Keywords:** Agaonidae, dioecy, *Ficus*, frugivores, seed dispersal

## Abstract

Dioecy allows separation of female and male functions and therefore facilitates separate co‐evolutionary pathways with pollinators and seed dispersers. In monoecious figs, pollinators' offspring develop inside the syconium by consuming some of the seeds. Flower‐stage syconia must attract pollinators, then ripen and attract seed dispersers. In dioecious figs, male (“gall”) figs produce pollen but not viable seeds, as the pollinators' larvae eat all seeds, while female (“seed”) figs produce mostly viable seeds, as pollinators cannot oviposit in the ovules. Hence, gall and seed figs are under selection to attract pollinators, but only seed figs must attract seed dispersers. We test the hypothesis that seed and gall syconia at the flower stage will be similar, while at the fruiting stage they will differ. Likewise, monoecious syconia will be more similar to seed than gall figs because they must attract both pollinators and seed dispersers. We quantified syconium characteristics for 24 dioecious and 11 monoecious fig species and recorded frugivore visits. We show that seed and gall syconia are similar at the flower stage but differ at the fruit stage; monoecious syconia are more similar to seed syconia than they are to gall syconia; seed and gall syconia differentiate through their ontogeny from flower to fruit stages; and frugivores visit more monoecious and seed syconia than gall syconia. We suggest that similarity at the flower stage likely enhances pollination in both seed and gall figs and that differentiation after pollination likely enhances attractiveness to seed dispersers of syconia containing viable seeds. These ontogenetic differences between monoecious and dioecious species provide evidence of divergent responses to selection by pollinators and seed dispersers.

## Graphical abstract

Dioecious figs offer an excellent opportunity to test the importance of mutualists to select for flower and fruit‐stage traits. At the flower stage, both male and females need to attract pollinators, but at the fruit stage only females need to attract seed disperser, as males do not bear viable seeds. We found that figs of both sexes indeed share traits at the flower stage but differentiate at the fruit stage, which is concordant with the hypothesis that mutualists select for flower and fruit appearance in the genus.

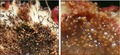

## 1 INTRODUCTION

Angiosperms are the most successful group of land plants, as they dominate most terrestrial habitats. Their success is due, at least partially, to their mutualistic interactions with pollinators and seed dispersers (Eriksson & Bremer, 1992). Angiosperm reproduction takes many forms that require different behaviors from mutualists. Dioecy, although rare, has evolved repeatedly in the evolutionary history of both angiosperms and gymnosperms, which has generated much interest in its advantages and potential limitations (Givnish, 1980; Käfer, Marais, & Pannell, 2017; Renner, 2014; Renner & Ricklefs, 1995). A major advantage of dioecy is that it allows separation of female and male functions, providing opportunities for more independent responses to selection by mutualists (Gleiser et al., 2019; Patel & McKey, 1998). This benefit of dioecy is usually framed with respect to pollination (i.e., male and female floral structures), but in a very unique system, *Ficus* (Moraceae), it also applies to the next step of angiosperm reproduction, seed dispersal (Dumont & O'Neal, 2004; Lambert, 1992; Weiblen, Lomascolo, Oono, & Dumont, 2010).

In *Ficus*, adaptations associated with dioecy are not straightforward because traits that are advantageous for pollination may be disadvantageous for seed dispersal, and vice versa. For example, flower‐bearing syconia of dioecious *Ficus* need to be similar between sexes, because both sexes need to attract the same pollinators, but dissimilar at the “fruiting” or seed dispersal stage, as only female syconia must attract seed dispersers (see below). Here we provide evidence that *Ficus* species have responded to opposing selection pressures between sexes and between stages of reproduction. Before presenting five predictions, we describe the relevant life history of monoecious and dioecious figs.

**Figure 1:**
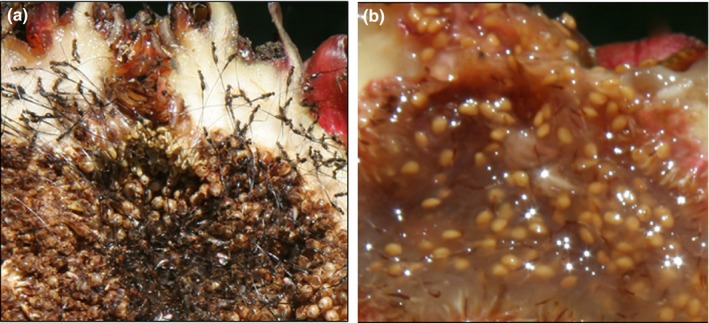
Comparison between a gall (a) and seed (b) fig of *Ficus dammaropsis* at the “mature” fruit stage. In the gall fig, adult female wasps have emerged from the endocarpus in the gall fig and are ready to emerge (note the long ovipositor, typical of fig‐pollinating Agaonid wasps). In the seed fig, no wasps develop, and the juicy interior of the fruit has only viable seeds, ready to be eaten by frugivores and dispersed

**Figure 2:**
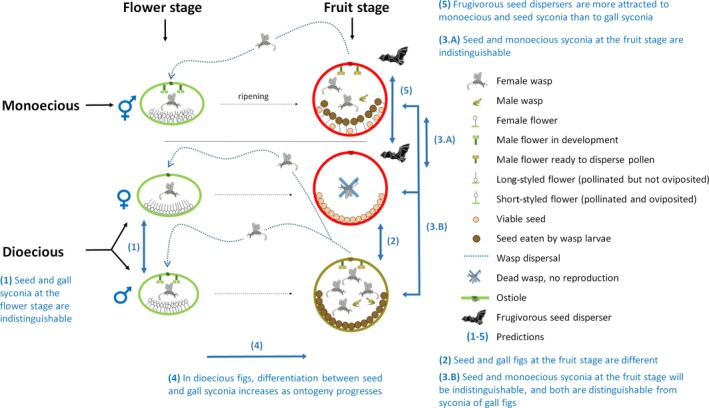
Life cycle of the pollinating fig wasp in monoecious and dioecious syconia. In monoecious species (top row) at the flower stage (left column), pollen‐loaded, fertilized female wasps enter the closed syconium, pollinate female flowers, and oviposit in some of them. Wasp larvae feed on developing seeds, emerge as adults, and copulate within the syconium. Fertilized females collect pollen from male flowers and leave the syconium through a hole drilled by male wasps just before the fig ripens to find another receptive fig of the same species, where their life cycle starts again. Pollinated flowers produce viable seeds that are animal‐dispersed when the syconium ripens. Male wasps die without ever leaving the syconium. In male, or “gall” syconia of dioecious figs, pollen‐loaded fertilized female wasps enter the syconium and oviposit in every flower they pollinate; essentially, all developing seeds are consumed by wasp larvae, so the gall syconia generally contain no viable seeds. Therefore, they should not be under selection to attract seed dispersers. In female, or “seed” syconia, pollen‐loaded, fertilized female wasps enter syconia and pollinate the flowers, but cannot oviposit in the long‐styled flowers. Female wasps die within syconia without reproducing. Seed figs produce viable seeds that benefit from seed dispersal and are therefore under selection to attract seed dispersers. Also illustrated are the five predictions tested in this study. The life stage and sex to which each prediction applies is marked with a blue arrow and its corresponding number

In the genus *Ficus*, monoecy is ancestral (Rønsted, Weiblen, Clement, Zerega, & Savolainen, 2008; Weiblen, 2000). Dioecy occurs in a little over half of all *Ficus* species and is restricted to the paleotropics (Berg, 1989). All figs have an extremely tight interaction with pollinators as, in general, each species of fig is predominantly pollinated by one species of wasp in the family Agaonidae (for exceptions, see Cook & Rasplus, 2003; Weiblen, 2002). In monoecious fig species, pollination is effected by pollen‐loaded, fertilized female wasps, which enter the closed syconium through a hole called the ostiole, tightly closed by overlapping involucral bracts (Datwyler & Weiblen, 2004), pollinate female flowers inside and oviposit in the ovules of some of the flowers. Wasp larvae feed on developing seeds. After completing development, wasps emerge through the seed coat and copulate within the syconium. Fertilized females collect pollen from male flowers and leave the syconium through a hole drilled by male wasps to find another receptive fig, where their life cycle starts again. Flowers pollinated by the incoming wasps produce viable seeds that are animal‐dispersed when the syconium develops into a “fruit” (Figures 1 and 2). Male wasps are wingless and hence never disperse from the syconium in which they emerged (Janzen, 1979; Weiblen, 2000). In an evolutionary sense, monoecious figs “pay” for viable seeds with seeds consumed by their pollinators.

In dioecious species, male and female functions are separated in different trees. In some trees, pollen‐loaded fertilized female wasps enter syconia and oviposit in every flower they pollinate; seeds do not develop as wasp larvae develop in their place. Consequently, syconia at the mature fruit stage contain practically no viable seeds. Those trees are technically monoecious, but only the male function is successful. Hence, they are commonly called male figs, or “gall” figs, because they pass their genes to the next generation only via male gametes, dispersed in the pollen collected and distributed by the female wasps (Weiblen, 2002) (Figures 1 and 2). Given that they essentially never produce viable seeds, gall figs are not under selection to attract seed dispersers. They “pay” for pollen dispersal with tissue that would have developed into mature seeds. In “female” trees, female wasps enter syconia and pollinate the flowers. However, they are unable to oviposit because the flowers have styles longer than the wasps' ovipositor; hence, female wasps die within syconia without leaving any descendants. Syconia on female trees are a deadly trap for the pollinating wasps, yet they produce viable seeds that benefit from seed dispersal (Figures 1 and 2). Female figs are called “seed” figs. In an evolutionary sense, seed figs pay nothing for pollination—they "cheat." They are, however, under selection to attract female wasps at the flowering stage and later to provide a nutritious reward to seed dispersers.

Monoecious and seed figs are dispersed by many types of frugivores (Shanahan, So, Gompton, & Gorlett, 2001), and syconia have evolved to attract seed dispersers in a variety of ways (Lomascolo, Levey, Kimball, Bolker, & Alborn, 2010). As described above, gall figs are presumably the exception because they contain no viable seeds. Perhaps more important, consumption of gall syconia would jeopardize the pollination mutualism, as all pollinators of dioecious figs require gall syconia for reproduction.

These complexities of reproductive natural history led Lambert (1992) and Dumont, Weiblen, and Winkelmann (2004) to propose a conceptual framework about selective pressures on different types of figs. Most straightforward, all figs—gall, seed, and monoecious—are under similar selection at the flower stage to attract Agaonid wasps. Postpollination (hereafter, at the fruit stage), only seed figs and monoecious figs would be expected to invest in traits that attract and reward seed dispersers. Gall figs should not invest in such traits; to the contrary, they should be structured to avoid consumption at all stages. Extending this rationale a step further, Lambert (1992) and Dumont et al. (2004) hypothesized that female plants should very closely mimic male plants at the flower stage to counteract strong selective pressure among female wasps to differentiate between “safe” gall syconia and “death trap” seed syconia. Yet, Grafen and Godfray (1991) propose that benefits of mimicry at the flower stage are reciprocal for seed and gall figs. It may be argued that gall figs also benefit from similarity because gall figs depend on emerging female wasps successfully finding and pollinating seed figs, as it is the only way in which the genes of gall figs will be represented in the next generation. Pollen‐loaded female wasps that enter another gall fig will pollinate the gall flowers, but no seeds will develop and, hence, genes from the pollen donor will not pass to the next generation.

Indeed, it has been shown that intersexual mimicry in volatile organic compounds (VOC), responsible for flower and fruit odor, does occur in at least some fig species whose gall and seed figs flower synchronously (Hossaert‐McKey et al., 2016). The role of selective pressure by pollinating wasps is further supported by the same authors' observation that mimicry does not occur in species whose gall and seed figs do not flower synchronously. Moreover, the VOC profile of fruit‐stage seed syconia is clearly different from that of gall syconia in a species of figs dispersed by mammals, which have keen olfaction (Borges, Bessière, & Hossaert‐McKey, 2008). In the same study, species dispersed by birds (which have generally poor olfaction) did not differ in VOCs between gall and seed figs, suggesting another stage—the fruit stage—at which figs can evolutionarily respond to differences in mutualists. Volatiles, however, may not tell the complete story. Although frugivores seem to prefer seed over gall figs (Chen et al., 2017; Lambert, 1992), the evidence is mostly anecdotal and at least some frugivorous bats do find and consume figs that lack the traits typical of mammal‐dispersed species, including fruit odor (Lomascolo et al., 2010).

We suggest a thorough test of Lambert's (1992) and Dumont et al.'s (2004) conceptual framework is lacking. Such a test would examine multiple monoecious and dioecious species at the flower and fruit stages and would integrate several traits known to influence fig choice by pollinators and frugivores with quantitative measures of frugivore consumption among syconia types.

Previous tests of the framework's predictions have yielded mixed support (Chen et al., 2017; Dumont et al., 2004; Lambert, 1992; Weiblen et al., 2010). We extend those studies by testing a series of predictions with data on traits known to be used by pollinators and frugivores to find resources and by standardized observations of feeding activity at fruiting figs. At the flower stage, we analyze syconia color, odor, and size because these traits have been shown to affect pollinator attraction (Dumont et al., 2004; Patel, Anstett, Hossaert‐McKey, & Kjellberg, 1995). At the fruit stage, we add contrast against the background, pulp softness, and length of the peduncle (a structure that exposes the syconium away from the branch and trunk, thereby increasing accessibility to frugivores), which are also reported to influence frugivore attraction (Dumont et al., 2004; Lambert, 1992; Lomascolo et al., 2010; Schmidt, Schaefer, & Winkler, 2004). We propose five predictions. Because we could not collect data on all stages of maturity for all species included in this study, we test these predictions with the most appropriate set of species in each case. Our predictions, illustrated in Figure 2, are as follows: (a) syconia of seed and gall figs at the flower stage will be indistinguishable in color, odor, and size; (b) syconia of seed figs will be dissimilar to syconia of gall figs at the fruit stage; (c) syconia of seed and monoecious figs at the fruit stage will be more similar to each other in color, odor, size, softness, and exposure away from branches and trunk (peduncle length), than they are to gall figs; (d) differentiation in all traits should increase with ontogeny between seed and gall syconia of the same species, from the flower stage to the unripe and ripe fruit stages; and (e) frugivores should visit monoecious and female fig trees more often than male fig trees.

## 2 MATERIALS AND METHODS

### 2.1 Study site and general specifications

We collected data between September 2004 and December 2005 in a lowland forest in Madang Province, Papua New Guinea, at Ohu Village (145°E, 5°S). To obtain data on color, contrast against the background, odor, size, softness, and peduncle length for seed and gall syconia of each species, we averaged records from 3 to 8 individual trees per species, per sex, and per stage of maturity. For each tree, between one and 20 syconia were measured and averaged for all traits. All measurements were taken separately for syconia at the flower and at the fruit stage.

Syconia were considered to be at the flower stage when we saw multiple fig wasps around the ostiole. In some cases, we even saw female wasps entering through the ostiole, a confirmation that the syconium was receptive. The fruit stage in gall syconia was determined by a hole in the ostiole, indicating that female wasps had already emerged and, hence, completion of the pollination cycle. For female syconia, we used several variables that characterize fruit maturation in most fleshy fruited species: changes in color, softness, and size in comparison with immature syconia. Syconia also had to detach easily from the tree to be considered ripe. The species included in this study are listed in Tables 2 (flower stage) and 3 (fruit stage).

### 2.2 Fruit and flower traits

#### 2.2.1 Color

We quantified syconium color using a USB2000 portable spectrometer (Ocean Optics, Inc) and a PX‐2 Pulsed Xenon light source, which took reliable readings between 300 and 740 nm. This range includes wavelengths visible by humans and ultraviolet wavelengths. We scanned syconia using a sensor that had five optical fibers illuminating the target surface and a sixth fiber that returned the reflected light to the spectrometer. The scanning angle was fixed at 45° by using a black metal stand with a hole at that angle. The metal stand also blocked external light. We fastened a non‐UV‐filtering microscope slide to the opening of the hole where the sensor was introduced to keep constant the distance between the end of the sensor and the syconium. To obtain an average reflectance spectrum for a given syconium, we scanned it three times in three different spots on the syconium. We calculated reflectance as the proportion of a certified white Spectralon Diffuse Reflectance Standard (Labsphere). All spectra for each species were averaged every 5 nm and further smoothed with a smoothing function in pavo, an R package for quantifying color (Maia, Eliason, Bitton, Doucet, & Shawkey, 2013; R Core Team, 2018). We obtained a quantitative measure of syconium color for each species with this same R package (of all the options included in pavo, we chose those that we specify in parenthesis), including hue, which is the wavelength of maximum reflectance (H1), mean relative brightness across the entire spectrum (B2), and chroma, which represents the saturation of the spectrum (S8) and is calculated as the wavelength of maximum reflectance minus the wavelength of minimum reflectance, divided by brightness (B2) (Maia et al., 2013; Ordano et al., 2017).

We calculated color contrasts as the Euclidean distance between the color of a syconium and the color of the structure against which it is seen by a mutualist, which consisted of leaves, bark or nearby unripe fruits when syconia were at the fruit stage, depending on the species. We normalized spectra to the same brightness by dividing the reflectance at each wavelength by the total reflectance for each species. This represents contrast due to the color of the syconium and not to brightness, and is called chromatic contrast (Schmidt et al., 2004).

$$D=\sqrt{\sum {\left[\text{Qf}(\lambda )-\text{Qb}(\lambda )\right]}^{2}}$$

Qf is the color spectrum of the syconium and Qb is the color spectrum of the background structure; *λ* is the wavelength in nm and the sum corresponds to the complete spectrum, 300–740 nm.

#### 2.2.2 Volatile compounds

To quantify odor, we collected syconia in the field and extracted the volatile compounds in the laboratory. In situ extraction of volatiles was not possible because plants were found in some very remote sites and some species were 30 m‐tall trees, which had to be carefully climbed to collect syconia. To ensure equal treatment of all species and both sexes, syconia were brought back to the laboratory, where volatile extraction started no more than 3 hr after collection. Syconia were placed inside 1‐gallon Reynolds oven baking bags that had a carbon filter at one end to clean air entering the bag, and a filter containing 50 mg of Super Q^®^ (80/100 mesh, Alltech Associates) to adsorb syconia volatiles in air exiting the bag. The filter was connected to a vacuum pump (Welch, model no. 2522B‐01), which sucked the air through the bag. All odor collections were run for four continuous hours. After odor collection, each filter was wrapped in aluminum foil and saved in a vial until it could be sent to the Chemistry Research Unit of the USDA in Gainesville, Florida, USA. To extract syconia volatiles from the Super Q filters, we passed 150 µl of methylene chloride through the filters to elute the trapped compounds. Extracts were stored at −80ºC prior to analysis. Gas chromatography (GC) analyses were performed on a Hewlett‐Packard (HP) 6890 gas chromatograph with He carrier gas at a constant flow of 30 cm/min. Samples were introduced by splitless injection at 240°C (injection volume 2 µl and split opened after 1 min.). The GC was equipped with a HP‐5MS column (5% phenyl methyl siloxane; 30 m long, 250 µm i.d., 0.25 µm film thickness) that was temperature programmed from 35°C (1 min hold) at 10°C/min to 230°C (hold for 5 min). The GC was coupled to a HP 5973 quadruple‐type mass selective detector with transfer line, source, and quadruple temperatures of 230, 230, and 150°C, respectively. Electron impact data were collected at a mass range of 35–400 amu and ionization energy of 70 eV.

Only the total amount of volatile compounds was used as a measure of how odorous the syconia were as, due to budget constraints, we were not able to run all samples through the mass spectrometer to identify the volatiles of all the species, sexes, and stages of maturity. We standardized the total amount of volatiles contained in a given sample by dividing it by the total surface area of the syconium used to collect that sample. Total surface area was calculated using the mathematical formula for the surface area of a sphere, using the average diameter of the syconia of each species, and multiplying this number by the number of syconia included in the sample. We acknowledge that some syconia were not perfectly spherical, but shape variation had little impact on our standardized estimates of total volatiles.

#### 2.2.3 Syconium size, softness, and peduncle length

For syconium size, we measured diameter at the widest point with a caliper to the nearest 0.5 mm and used it as an estimate of overall size. For softness, an arbitrary scale between 1 (hardest, syconium surface did not indent when pressed between thumb and index finger) to 4 (softer, syconium surface easily indented when pressed identically) was developed by the authors and measured by the same person throughout the study (Lomascolo et al., 2010). Peduncle length (±0.5 mm) was measured between the base of the syconium and the point where the peduncle attached to the branch or trunk.

### 2.3 Visitation to fig trees

We quantified visits to monoecious, seed, and gall figs using two video cameras (Sony DCR‐HC40) positioned 3–5 m from a fruiting tree and recording simultaneously. Videotaping started at 6 a.m. and ended at 10:30 a.m., and then again from 6:30 p.m. until 11:00 p.m., which allowed us to detect both diurnal and nocturnal frugivores. Nocturnal recording was done using an infrared light (Sony HVL‐IRH2). We aimed one camera at the ground to record terrestrial frugivores and one at either a branch or the trunk, depending on where most ripe figs occurred on the tree. We recorded 68 individual seed figs (1,077.3 hr) of 28 species (Table 1) and 20 gall figs (339.4 hr) of seven species (Table 1). Monoecious species were less common in our study site, so we were able to record four monoecious trees (68.8 hr) of four species (Table 1).

**Table 1:** All fig species of different sexes that were videotaped to record visitation by dispersers

Species of *Ficus*	Individuals per sex	Individual code	Disperser	Sex	Time of videotaping	Total minutes of videotaping
*F. edelfeltii*	1	EDE1BA	None	Monoecious	AM	292
	1	EDE1BP	Fruit bat	Monoecious	PM	300
*F. hesperidiiformis*	2	HES1BA	Bird	Monoecious	AM	478
	2	HES1BP	Fruit bat	Monoecious	PM	472
*F. hombroniana*	3	HOM1BA	None	Monoecious	AM	788
	3	HOM1BP	Fruit bat	Monoecious	PM	749
*F. subtrinervia*	4	SUV2FA	None	Monoecious	AM	541
	4	SUV2FP	None	Monoecious	PM	509
*F. spA*	1	A5FA	None	Seed	AM	521
	1	A5FP	Bandicoot	Seed	PM	542
	2	A6FA	None	Seed	AM	537
	2	A6FP	None	Seed	PM	412
*F. adenosperma*	3	ADE8FA	None	Seed	AM	538
	3	ADE8FP	None	Seed	PM	516
	4	ADE9FA	None	Seed	AM	506
	4	ADE9FP	None	Seed	PM	519
*F. arfakensis*	5	ARF10FA	None	Seed	AM	532
	5	ARF10FP	Fruit bat	Seed	PM	474
	6	ARF1FA	None	Seed	AM	266
	6	ARF1FP	None	Seed	PM	234
	7	ARF2FA	None	Seed	AM	173
	7	ARF2FP	None	Seed	PM	236
	8	ARF9FA	None	Seed	AM	468
	8	ARF9FP	None	Seed	PM	498
*F. bernaysii*	9	BER7FA	None	Seed	AM	535
	9	BER7FP	None	Seed	PM	463
	10	BER9FA	None	Seed	AM	486
	10	BER9FP	Fruit bat	Seed	PM	498
*F. botryocarpa*	11	BOT11FA	None	Seed	AM	532
	11	BOT11FP	None	Seed	PM	517
	12	BOT3FA	None	Seed	AM	508
	12	BOT3FP	None	Seed	PM	417
*F. conocephalifolia*	13	COF6FA	None	Seed	AM	469
	13	COF6FP	None	Seed	PM	252
	14	COF9FA	None	Seed	AM	644
	14	COF9FP	None	Seed	PM	532
*F. congesta*	15	CON10FA	None	Seed	AM	523
	15	CON10FP	Fruit bat	Seed	PM	268
	16	CON15FA	None	Seed	AM	536
	16	CON15FP	Fruit bat	Seed	PM	526
	17	CON1FA	None	Seed	AM	479
	17	CON1FP	Fruit bat	Seed	PM	733
*F. copiosa*	18	COP6FA	None	Seed	AM	554
	18	COP6FP	Fruit bat	Seed	PM	512
*F. dammaropsis*	19	DAM5FA	None	Seed	AM	550
	19	DAM5FP	None	Seed	PM	544
*F. hispidioides*	20	HIS10FP	None	Seed	PM	492
	21	HIS1FA	None	Seed	AM	525
	21	HIS1FP	None	Seed	PM	940
	22	HIS5FA	None	Seed	AM	506
	22	HIS5FP	None	Seed	PM	512
	23	HIS6FA	None	Seed	AM	387
	23	HIS6FP	Bat	Seed	PM	393
*F. itoana*	24	ITO1FA	None	Seed	AM	510
	24	ITO1FP	None	Seed	PM	544
	25	ITO3FA	None	Seed	AM	539
	25	ITO3FP	None	Seed	PM	500
	26	ITO9FA	None	Seed	AM	538
	26	ITO9FP	None	Seed	PM	538
*F. spM*	27	M2FA	None	Seed	AM	473
	28	M2FA'	None	Seed	AM	525
	27	M2FP	None	Seed	PM	447
	28	M2FP'	None	Seed	PM	531
*F. macrorhynca*	29	MAC1FA	Bird	Seed	AM	526
*F. melanocarpa*	30	MEL1FA	None	Seed	AM	431
	30	MEL1FP	None	Seed	PM	504
*F. mollior*	31	MOL10FA	None	Seed	AM	407
	31	MOL10FP	None	Seed	PM	402
	32	MOL2FA	None	Seed	AM	465
	32	MOL2FP	None	Seed	PM	505
	33	MOL9FA	None	Seed	AM	560
	33	MOL9FP	None	Seed	PM	410
*F. morobensis*	34	MOR1FA	None	Seed	AM	265
	34	MOR1FP	None	Seed	PM	179
	35	MOR2FA	None	Seed	AM	462
	35	MOR2FP	Fruit bat	Seed	PM	533
	36	MOR7FA	None	Seed	AM	1,047
	36	MOR7FP	Fruit bat	Seed	PM	1,043
	37	MOR8FA	None	Seed	AM	541
	37	MOR8FP	None	Seed	PM	517
*F. pachyrrachis*	38	PAC11FA	None	Seed	AM	515
	38	PAC11FP	None	Seed	PM	269
	39	PAC12FA	Bird	Seed	AM	517
	39	PAC12FP	None	Seed	PM	476
	40	PAR10FA	None	Seed	AM	507
	40	PAR10FP	None	Seed	PM	412
	41	PAR1FA	None	Seed	AM	251
	41	PAR1FP	None	Seed	PM	162
*F. phaeosyce*	42	PHA4FA	None	Seed	AM	526
	42	PHA4FP	None	Seed	PM	476
*F. spPRI*	43	PRI1FA	None	Seed	AM	420
	43	PRI1FP	None	Seed	PM	495
*F. pungens*	44	PUN16FA	None	Seed	AM	542
	45	PUN17FA	None	Seed	AM	528
	45	PUN17FP	Bandicoot, bat	Seed	PM	500
	46	PUN1FA	None	Seed	AM	442
	46	PUN1FP	Fruit bat	Seed	PM	511
	47	PUN2FA	Bird	Seed	AM	529
	48	PUN2FA'	None	Seed	AM	520
	47	PUN2FP	Bandicoot	Seed	PM	247
	48	PUN2FP'	None	Seed	PM	499
*F. semivestita*	49	SEM1FP	Fruit bat	Seed	PM	477
*F. septica*	50	SEP7FA	None	Seed	AM	547
	50	SEP7FP	None	Seed	PM	529
*F. subulata*	51	SUB5FA	None	Seed	AM	1,021
	51	SUB5FP	None	Seed	PM	538
	52	SUB7FA	None	Seed	AM	508
	52	SUB7FP	Fruit bat	Seed	PM	482
	53	SUB8FA	None	Seed	AM	512
	53	SUB8FP	None	Seed	PM	514
*F. ternatana*	54	TER1FA	None	Seed	AM	504
	54	TER1FP	None	Seed	PM	545
*F. trachypison*	55	TRA3FA	Bird	Seed	AM	509
	55	TRA3FP	None	Seed	PM	413
	56	TRA4FA	None	Seed	AM	492
	56	TRA4FP	None	Seed	PM	500
*F. variegata*	57	VAR10FA	None	Seed	AM	555
	57	VAR10FP	Fruit bat	Seed	PM	544
	58	VAR11FA	None	Seed	AM	544
	58	VAR11FP	Fruit bat	Seed	PM	473
	59	VAR8FA	None	Seed	AM	482
	60	VAR8FA'	None	Seed	AM	426
	59	VAR8FP	Fruit bat	Seed	PM	366
	60	VAR8FP'	None	Seed	PM	498
*F. virgata*	61	VIR1FA	None	Seed	AM	493
	61	VIR1FP	None	Seed	PM	490
	62	VIR6FA	None	Seed	AM	499
	62	VIR6FP	Fruit bat	Seed	PM	494
	63	VIR7FA	None	Seed	AM	534
	63	VIR7FP	None	Seed	PM	563
	64	VIR8FA	None	Seed	AM	516
	64	VIR8FP	None	Seed	PM	535
*F. wassa*	65	WAS10FA	None	Seed	AM	462
	65	WAS10FP	None	Seed	PM	543
	66	WAS1FA	None	Seed	AM	278
	67	WAS2FA	None	Seed	AM	377
	67	WAS2FP	None	Seed	PM	211
	68	WAS9FA	None	Seed	AM	551
	68	WAS9FP	None	Seed	PM	540
*F. congesta*	1	CON10MA	None	Gall	AM	481
	1	CON10MP	None	Gall	PM	475
	2	CON13MA	None	Gall	AM	536
	2	CON13MP	None	Gall	PM	525
	3	CON15MA	None	Gall	AM	529
	3	CON15MP	None	Gall	PM	561
	4	CON16MA	None	Gall	AM	520
	4	CON16MP	None	Gall	PM	533
*F. hispidioides*	5	HIS10MA	None	Gall	AM	519
	5	HIS10MP	None	Gall	PM	494
	6	HIS11MA	None	Gall	AM	522
	6	HIS11MP	None	Gall	PM	517
	7	HIS12MA	None	Gall	AM	539
	7	HIS12MP	None	Gall	PM	514
	8	HIS4MA	None	Gall	AM	466
	8	HIS4MP	None	Gall	PM	579
*F. morobensis*	9	MOR6MA	None	Gall	AM	537
	9	MOR6MP	None	Gall	PM	438
*F. pachyrrachis*	10	PAC13MA	None	Gall	AM	522
	10	PAC13MP	None	Gall	PM	492
	11	PAC14MA	None	Gall	AM	532
	11	PAC14MP	None	Gall	PM	506
	12	PAC8MA	None	Gall	AM	530
	12	PAC8MP	None	Gall	PM	481
	13	PAR4MA	None	Gall	AM	480
	13	PAR4MP	None	Gall	PM	505
*F. pungens*	14	PUN10MA	None	Gall	AM	515
	14	PUN10MP	None	Gall	PM	351
	15	PUN14MA	None	Gall	AM	536
	15	PUN14MP	None	Gall	PM	433
	16	PUN18MA	None	Gall	AM	525
	16	PUN18MP	None	Gall	PM	534
	17	PUN19MA	None	Gall	AM	510
	17	PUN19MP	None	Gall	PM	546
	18	PUN5MA	None	Gall	AM	748
	18	PUN5MP	None	Gall	PM	830
*F. septica*	19	SEP10MA	None	Gall	AM	441
	19	SEP10MP	None	Gall	PM	506
*F. virgata*	20	VIR6MA	None	Gall	AM	514
	20	VIR6MP	Bandicoot	Gall	PM	522

Note

Time of videotaping refers to AM: morning, between 6:00 and 10:30 a.m.; PM: evening, between 6:30 and 11:00 p.m. The column “Individuals per sex” refers to the number of individuals that were recorded. The same number denotes the same individual within each sex, as each individual was videotaped during the morning and in the evening, with very few exceptions.

### 2.4 Data analysis

#### 2.4.1 Difference in traits of syconia at different developmental stages

To test prediction 1, whether gall and seed figs are indistinguishable at the flower stage, we applied a generalized linear model (GLM)—specifically, a logit model with a binomial family error (Gelman & Hill, 2007; Piquer‐Rodríguez et al., 2018; Rojas, Vergara‐tabares, Valdez, Ponzio, & Peluc, 2019), with sex as the dependent variable and color (hue, brightness, and chroma, Endler, 1990), total amount of volatile compounds, and diameter as the predictor variables. For this test, we had complete data on six species of gall figs and seven seed figs (Table 2). To test prediction 2—that syconia from seed and gall figs will be distinguishable at the ripe fruit stage—we performed the same GLM between gall and seed figs (*n* = 24 species of each; Table 3), adding chromatic contrast against the background, pulp softness, and peduncle length as predictor variables because they are known to influence frugivore preference (Lomascolo et al., 2010; Schmidt & Schaefer, 2004) and likely have little influence on wasp behavior. To test prediction 3—that seed and monoecious figs at the ripe fruit stage are more similar to each other than they are to gall figs—we used the same predictor variables as for prediction 2 and performed two tests, hereafter 3A and 3B. Test 3A: At the ripe fruit stage, we performed a binomial logistic regression between seed (*n*_seed_ *= *24 species) and monoecious figs (*n*_mono_ *= *11 species) (Table 3). Test 3B: We performed a GML of the multinomial logit type, comparing seed (*n*_seed_ *= *24 species), gall (*n*_gall_ *= *24 species) and monoecious (*n*_mono_ *= *11 species) figs with the same variables and sets of species as the previous analyses (Table 3).

**Table 2:** Species of *Ficus* included in the logistic regression aiming at testing the prediction that seed and gall figs are indistinguishable at the flower stage, as both types of figs are under selection to attract pollinating wasps

Species	Sex	B2	H1	S8	Softness	Diameter (cm)	Total volatiles (mV/mm^2^)	Morphology and color			Odor		
								# individuals	Mean # of syconia	STD # of syconia	# individuals	Mean # of syconia	STD # of syconia
*F. adelpha*	Female	4.64	550	2.15	2	13.68	0	1	5	N/A	1	20	N/A
*F. arfakensis*	Female	1.78	600	2.03	2.12	12.72	10.93	1	5	N/A	1	5	N/A
*F. botryocarpa*	Female	3.28	545	2.32	1	19.3	16.11	2	4.5	0.71	2	8.5	3.54
*F. botryocarpa*	Male	4.83	550	2.11	1	19.8	6.8	1	5	N/A	1	6	N/A
*F. congesta*	Female	5.84	550	2.19	1.17	23.28	14.45	2	5	0	2	10.5	0.71
*F. congesta*	Male	5.18	550	2.34	2.12	22.94	33.42	2	5	0	2	10	2.83
*F. hispidoides*	Male	6.72	550	2.18	1.1	30.59	44.63	4	4.25	1.5	4	4.25	1.71
*F. nodosa*	Female	9.39	620	1.65	1.5	22.43	316.93	1	4	N/A	1	4	N/A
*F. pachyrrachis*	Female	2.07	615	2.38	1	44.15	36.02	1	2	N/A	1	6	N/A
*F. pungens*	Female	9.59	580	1.78	2	5.1	7.37	2	5	0	2	212	195.16
*F. pungens*	Male	9.16	610	1.56	3	4.3	19.26	2	5	0	2	315	21.21
*F. septica*	Male	3.55	545	3.5	1.5	17.66	21.02	1	5	N/A	1	11	N/A
*F. wassa*	Male	11.05	555	1.59	2	6.95	0	2	5	0	2	15.5	14.85

Note

The variables included in the analysis are listed in columns 3–8 and are syconium brightness (amount of light reflected by the fruit, B2), hue (wavelength of peak reflectance, generally interpreted as the color of the syconium, H1), saturation (how “pure” a color looks, represented by the slope of the curve approaching peak reflectance, S8), syconium softness (a measure of the range from dry, fibrous syconia, to soft, moist and fleshy), diameter at the widest point, and total amount of volatiles quantified by gas chromatography. The last six columns represent the number of individuals of each species included in the analyses of morphology, color, and extraction of volatile compounds, as well as the mean and standard deviation of number of syconia per individual.

**Table 3:** Species of *Ficus* included in the logistic regressions to look at how similar are monoecious, seed, and gall syconia at the ripe fruit stage

Species	Sex	B2	H1	S8	softness	Diameter (cm)	Total volatiles (mV/mm^2^)	Peduncle length (mm)	Contrast	Morphology and color			Odor		
										# of individuals	Mean # of syconia	STD # of syconia	# of individuals	Mean # of syconia	STD # of syconia
*F. adenosperma*	Female	4.77	555	1.87	3	14.59	51.59	5.48	90.67	2	5	0	2	14.5	6.36
*F. adelpha*	Female	8.28	615	1.59	2.67	19.45	477.46	24.62	35.02	3	4	1	3	7.67	7.23
*F. arfakensis*	Female	2.34	655	2.17	3.28	18.83	217.41	7.54	23.64	4	5	0	4	16.75	5.32
*F. bernaysii*	Female	3.61	690	1.6	2.95	16.71	190.29	44.6	30.35	3	5	0	2	27.5	26.16
*F. botryocarpa*	Female	3.88	555	1.54	2.7	32.29	219.44	26.47	28.29	5	4.6	0.55	5	5.8	1.92
*F. conocephalifolia*	Female	3.06	685	3.18	3.38	31.47	3.29	13.53	55.58	6	1.83	1.6	3	3	2.65
*F. congesta*	Female	5.98	570	1.57	3.08	40.75	47.49	15.55	32.07	8	4.13	1.23	8	5.63	3.02
*F. copiosa*	Female	4.63	625	1.89	2.93	46.26	61.2	40.9	65.17	4	4	0	3	3	1
*F. dammaropsis*	Female	7.47	645	2.09	3	69.49	45.99	0	82.59	3	1.67	0.58	3	1.67	0.58
*F. gul*	Female	2.96	640	2.05	4	9.05	56.52	8.31	38.08	2	5	0	2	22.5	3.54
*F. hispidioides*	Female	7.53	620	1.61	3.25	52.13	4.46	13.51	25.27	3	3.33	1.53	3	3.33	1.53
*F. itoana*	Female	5.05	675	2.37	3.13	46.69	120.54	33.83	46.7	4	1.75	0.5	4	1.75	0.5
*F. macrorhynca*	Female	3.09	690	2.88	4	7.38	0	3	63.03	1	1	N/A	1	15	N/A
*F. mollior*	Female	3.93	610	1.68	3.13	17.21	30.45	11.95	78.57	4	5	0	3	32	12.53
*F. morobensis*	Female	6.36	645	1.99	2.83	38.34	395.31	92.33	9.82	3	4	2	4	3.75	2.75
*F. nodosa*	Female	5.04	565	1.93	3.11	34.18	69.85	23.97	82.19	3	3.33	1.53	3	3.33	1.53
*F. pachyrrachis*	Female	2.29	625	2.11	2.88	59.37	120.25	34.89	21.01	5	3	1	4	2.75	0.96
*F. phaeosyce*	Female	3.8	680	1.64	3.73	8.36	19.67	1.27	79.22	3	5	0	3	57.67	45.18
*F. pungens*	Female	2.52	685	2.99	3.2	8.98	91.41	5.55	41.65	5	10.6	6.27	5	83	66.8
*F. septica*	Female	4.56	550	2.32	2.83	31.01	47.7	11.14	82.97	3	4	1.73	3	7	3
*F. subulata*	Female	3.36	685	2.77	3.58	11.28	10.2	5.27	117.57	6	4.33	1.63	3	26.33	10.12
*F. variegata*	Female	4.64	565	1.93	3.07	37.73	31.54	34.88	118.11	5	3.2	2.05	3	7.67	7.64
*F. virgata*	Female	2	685	3.5	3.67	12.27	3.69	0	115.53	3	4.33	1.16	3	53.67	61.5
*F. wassa*	Female	2.27	685	3.22	3.68	16.29	2.38	14.89	184.96	4	5	0	3	50	27.4
*F. adenosperma*	Male	5.96	555	1.65	2.53	10.9	13.55	7.6	99.07	2	5	0	2	27	19.8
*F. adelpha*	Male	4.47	555	1.99	3	25.26	3.41	15.36	22.88	3	5	0	3	14	8.54
*F. arfakensis*	Male	3.77	635	1.76	2.63	20.07	0.47	8.44	25.55	3	5	0	3	32.67	28.57
*F. bernaysii*	Male	3.05	655	1.47	2.67	21.25	6.61	52.25	27.68	3	4.33	1.15	4	18.25	14.52
*F. botryocarpa*	Male	8.25	615	1.2	2.35	36.92	19.29	15.07	28.92	5	4.8	0.45	4	6.75	2.22
*F. conocephalifolia*	Male	4.94	675	2.18	2.81	36.64	24.98	15.94	47.39	4	1.5	0.58	3	1.33	0.58
*F. congesta*	Male	6.4	615	1.52	3	41.1	1.46	17.52	78.31	1	4	1	3	4.33	1.53
*F. copiosa*	Male	8.54	625	1.44	2.81	45.78	39.62	53.46	71.74	2	4.5	0.71	2	5	1.41
*F. dammaropsis*	Male	5.43	675	2.52	3	71.31	90.25	5.25	88.47	3	1.33	0.58	3	1.33	0.58
*F. gul*	Male	6.95	570	1.35	3	13.85	19.35	7.63	72.56	1	5	N/A	1	5	N/A
*F. hispidioides*	Male	6.9	555	1.59	2.63	53.8	42.74	25.06	19.07	8	3	1.93	8	3	1.93
*F. itoana*	Male	7.35	645	2.48	3.17	58.88	11.84	52.91	33.21	2	4	1.41	2	3	0
*F. macrorhynca*	Male	7.01	640	2.24	3	9.66	0	3.5	79.05	1	5	N/A	1	19	N/A
*F. mollior*	Male	7.31	610	1.83	3	18.77	19.21	9.55	75.35	3	5	0	3	28	22
*F. morobensis*	Male	6.02	625	1.57	2.17	54.13	104.17	90	15	4	3.75	1.5	3	3.33	1.53
*F. nodosa*	Male	5.74	635	1.55	3	40.21	5.56	11.14	80.38	2	5	0	3	5	0
*F. pachyrrachis*	Male	4.98	630	1.85	2.13	58.95	11.7	19.51	10.88	4	2.5	0.58	3	2.33	0.58
*F. phaeosyce*	Male	6.28	685	1.71	3	7.71	2.3	0.42	74.69	3	5	0	3	111.67	160.58
*F. pungens*	Male	8.25	655	1.7	3	7.29	14.3	3.01	10.03	5	8	6.71	3	70	57.19
*F. septica*	Male	7.04	555	1.68	2.73	27.62	9.88	10.3	87.41	5	5.8	2.39	5	17.8	12.93
*F. subulata*	Male	9.55	620	1.76	2.97	10.63	0.89	3.83	113.06	3	5	0	1	89	N/A
*F. variegata*	Male	4.65	610	1.99	2.75	32.19	2.87	29.1	62.36	3	5	0	4	16.75	10.72
*F. virgata*	Male	12.75	625	2.1	3.25	11.89	2.09	0	131.63	2	5	0	2	22	21.21
*F. wassa*	Male	7.71	645	1.79	3.25	15.71	26.44	17.94	126.04	5	5	0	5	57.6	38.62
*F. benjamina*	Monoecious	5.57	690	2.82	3.5	9.78	0.39	0	75.08	2	5	0	2	27.5	26.16
*F. drupaceae*	Monoecious	1.25	700	5.94	3.5	17.2	1.73	0	62.96	1	5	N/A	1	26	N/A
*F. edelfeltii*	Monoecious	5.02	620	1.99	3	46.43	655	2.7	66.65	1	3	N/A	1	3	N/A
*F. elastica*	Monoecious	4.93	680	3.21	3	12.67	117.2	7.43	83.14	1	3	N/A	1	3	N/A
*F. hesperidiiformis*	Monoecious	3.5	685	1.64	2.2	45.27	2.52	23.83	81.49	2	4	1.41	2	3	0
*F. hombroniana*	Monoecious	2.72	655	2.35	3	25.09	171.39	13.02	88.14	2	5	0	2	32	16.97
*F. microcarpa*	Monoecious	2.44	525	1.62	4	8.23	273.18	3.4	105.88	3	5	0	3	119.67	4.51
*F. polyantha*	Monoecious	9.12	580	1.95	3	36.51	36.1	21.91	101.06	2	4	0	2	4	0
*F. prasinicarpa*	Monoecious	1.57	695	3.33	3.83	11.85	96.43	4.59	66.87	3	5	0	2	14.5	3.54
*F. subtrinervia*	Monoecious	5.21	675	2.52	3.25	11.75	9.83	0	102.7	3	5	0	3	31.33	33.62
*F. xylosice*	Monoecious	1.92	655	3.59	4	17.56	10.66	12.2	125.39	1	5	N/A	1	11	N/A

Note

We tested the predictions that monoecious and seed figs would be similar to each other, as they are both under selection to attract frugivorous seed dispersers. These two fig types would be different from gall figs, which do not produce viable seeds and, thus, are not under selection to attract seed dispersers. The variables included in the analysis are listed in columns 3–10 and are syconium brightness (amount of light reflected by the fruit, B2), hue (wavelength of peak reflectance, generally interpreted as the color of the syconium, H1), saturation (how “pure” a color looks, represented by the slope of the curve approaching peak reflectance, S8), syconium softness (a measure of the range because dry, fibrous syconia, to soft, moist and fleshy), diameter at the widest point, total amount of volatiles quantified by gas chromatography, length of the peduncle that attaches the fruit to a branch of trunk, and chromatic contrast against the structure against which the syconium was exposed (foliage or trunk). The last six columns represent the number of individuals of each species included in the analyses of morphology, color, and extraction of volatile compounds, as well as the mean and standard deviation of number of syconia per individual.

For all tests described above, we ran three models to determine how well each separated between the groups: one with color variables only, one with color and odor variables, and one with all variables for color, odor, and structure (Table 4). We first ran the model with all variables, recorded the AIC, and checked for the significance of each variable in differentiating between the groups. Second, based on AIC we identified the best model and performed a cross‐validation (Table 5). For cross‐validation, we randomly selected 60% of the data to use as a "training" set in developing a model. We then used the remaining 40% of the data to test the capacity of the model to classify new individuals into the categories of each test. We ran each of the tests 100 times to obtain an average confusion matrix and an average classification error, which is the average probability of cases from each group to be classified in its own group versus in the two other groups. The classification error indicates how well the model classified the new cases from the training set into their proper categories.

**Table 4:** Evaluation of models

Model description (dependent and independent variables)	AIC	McFadden's Pseudo *R* ^2^	Mean misclassification error	Variables included in the model (Significant variables in bold; **p < .01; *p < .05; ^ǂ^ *p* < 0.1)
1. Dependent: Gall versus Seed Flowers				
Independent				
Color	21.28**	0.26	0.399	Hue, saturation, brightness
Color + Odor	22.75	0.29		Hue, saturation, brightness, total amount of aromatic compounds
Color + Odor + Structural	24.45	0.306		Hue, saturation, brightness, total amount of aromatic compounds, diameter
2. Dependent: Gall versus Seed Fruits				
Independent				
Color	58.46	0.272		Hue, saturation, **brightness, chromatic contrast
Color + Odor	44.99	0.504		Hue, saturation, *brightness, chromatic contrast, *total amount of aromatic compounds
Color + Odor + Structural	30.98**	0.805	0.172	Hue, saturation, brightness, chromatic contrast, **^ǂ^total amount of aromatic compounds**, diameter, **^ǂ^pulp softness**, peduncle length
3. A. Dependent: Monoecious versus Seed Fruits				
Independent				
Color	47.67**	0.136	0.274	Hue, saturation, brightness, chromatic contrast
Color + Odor	46.47	0.209		Hue, saturation, brightness, chromatic contrast, total amount of aromatic compounds
Color + Odor + Structural	49.67	0.273		Hue, saturation, brightness, chromatic contrast, total amount of aromatic compounds, diameter, pulp softness, peduncle length
3. B. Dependent: Gall versus Seed Vs. Monoecious Fruits				
Independent				
Color	113.33	0.243		Hue, saturation, **brightness, chromatic contrast
Color + Odor	98.54**	0.395		Hue, saturation, *brightness, *chromatic contrast, **total amount of aromatic compounds
Color + Odor + Structural	99.89	0.482	0.391	Hue, saturation, *brightness, chromatic contrast, **total amount of aromatic compounds, diameter, *pulp softness, peduncle length

Note

The best models, based on AIC and simplicity (in cases where the difference in AIC was less than two), are marked with an asterisk. For those models, we calculated a mean misclassification error by averaging 100 runs of the model done with random subsets of 40% of the data (testing set). The model was built with the other 60% of the data (training set). We also report McFadden's pseudo *R* ^2^ as a different estimate of model fit. The last column provides more detail of the variables included in each model and indicates the level of significance of those with the most influence. Models are numbered according to the predictions that they test, as described in the main text and Figure 2.

**Table 5:** Mean confusion matrices resulting from cross‐validation tests of each of the models described in Table 4

1. Gall versus Seed—Flower stage			2. Gall versus Seed—Fruit stage		
	Seed	Gall		Seed	Gall
Seed	1.01	0.66	Seed	8.28	1.65
Gall	1.74	2.59	Gall	1.8	8.28

3.A. Monoecious versus seed—Fruit stage			3.B Gall versus Seed versus Monoecious—Fruit stage			
	Monoecious	Seed		Monoecious	Seed	Gall
Monoecious	1.77	1.11	Monoecious	1.4	2.65	0.65
Seed	2.72	8.4	Seed	1.56	5.72	1.66
			Gall	1.39	1.43	7.54

Note

Models compare (1) gall versus seed syconia at the flower stage, (2) gall versus seed syconia at the ripe fruit stage, (3) monoecious and seed syconia at the ripe fruit stage, and (4) monoecious, seed, and gall syconia at the ripe fruit stage. Predictors include color, odor, and structural variables. The only structural variable included in tests involving syconia at the flower stage is diameter. Structural variables included in tests involving syconia at the fruit stage are diameter, pulp softness, peduncle length (as seen in Table 4). Each matrix results from tests with training and test data sets, as in Table 4.

To address prediction 4—that female and male syconia of the same species will increasingly differentiate as ontogeny (syconium maturation) progresses—we picked the only three species (*Ficus botryocarpa, Ficus pungens,* and *Ficus congesta*) for which we had data for all stages of maturity (flower, immature, and mature fruits) for both seed and gall figs. We ran a Principal Components Analysis (PCA) to ordinate all species in a multivariate space defined by the following variables: hue, brightness, saturation, total volatile compounds, diameter, and pulp softness. We calculated the Euclidean distance for the first three principal components (PCs) between gall and seed figs of the same species at the different developmental stages (i.e., seed vs. gall syconia at the flower stage, immature fruit stage, and mature fruit stage). This distance represents the difference between seed and gall figs of the same species at the same stage of development, which we would expect to increase according to prediction 3. Because we had only three data points for each maturity stage for each sex (one set of three points for each of three species), statistical power was very low. We therefore compared the Euclidean distances qualitatively, intending the result to inform future studies.

#### 2.4.2 Differential visitation to seed and gall figs by frugivores

To test prediction 5——that frugivores visit monoecious and seed figs more often than gall figs——we used a chi‐squared test to compare the number of visited and not‐visited monoecious, seed, and gall trees. We applied William's correction due to the small sample sizes (McDonald, 2014). We also performed a separate analysis just for female and male trees, as it seemed a cleaner test given that they were the same species of both sexes. For this analysis, we applied a Yate's continuity correction (McDonald, 2014). Any frugivore that entered the tree was assumed to have been attracted to the tree and its presence was recorded as a visit, unless it was clearly observed for the entire visit and did not consume a fruit during that time. A tree was recorded as visited if at least one frugivore visited it.

## 3 RESULTS

### 3.1 Differentiation of gall and seed figs at the flower stage (prediction 1)

Gall and seed figs at the flower stage were largely indistinguishable, regardless of which variables were included in the logistic models (Table 4). The model that included only color variables had the lowest AIC value, which was more than two units lower than that of the full model, but less than two units different than the model with both color and odor variables. We calculated the mean confusion matrix for the model with lowest AIC, averaged over 100 runs (Table 5), and it showed that seed syconia were classified almost randomly as either seed or gall, while gall syconia were a bit more accurately classified as gall. Overall, however, the classification error, calculated as 1‐ (# of correct predictions/total observations), was quite high: 40% (averaged over 100 runs, Table 4). This result means that each time a new syconium of unknown sex at the flower stage is classified based on the variables included in the best model, it will be incorrectly classified 40% of the time. If gall and seed syconia were identical, the classification error would be 50% (i.e., random).

### 3.2 Differentiation of gall, seed, and monoecious figs at the fruit stage (predictions 2 and 3)

#### 3.2.1 Gall versus seed syconia (prediction 2)

In all three models comparing gall and seed syconia at the mature fruit stage, at least two variables significantly or marginally significantly differentiated between gall and seed syconia. The model with the lowest AIC included all the variables. The mean confusion matrix averaged over 100 runs (Table 5) showed that seed and gall syconia at the ripe fruit stage tended to be classified much more accurately into their correct category than seed and gall syconia at the flower stage (mean classification errors 17% and 40%, respectively).

#### 3.2.2 Seed versus monoecious versus gall syconia (prediction 3)

##### Test 3A

Seed and monoecious syconia at the ripe fruit stage did not differ significantly; no variable was a significant predictor in any of the three models. The model with the lowest AIC included color and odor variables, but no model had an AIC that was less than two units below that of the model with only color variables. We calculated the confusion matrix and misclassification error for the simplest model, which included only color variables. Misclassification error, 27%, was higher than that for gall and seed syconia (17%). The average confusion matrix shows that, although seed syconia are quite well classified, classification of monoecious syconia had a high error rate. Overall, these results indicate that monoecious and seed syconia at the ripe fruit stage are similar in appearance.

##### Test 3B

When comparing gall versus seed versus monoecious syconia with a multinomial regression, the categories were significantly differentiated by at least one of the variables, and up to three variables in some of the models (Table 4). Again, as predicted and in line with results from analyses reported above, the largest differences were between gall and the other two types of syconia, as the calculated probability of being misclassified was much lower for gall syconia than for ripe seed or monoecious syconia (77%, 51%, and 1%, respectively). The highest probability of misclassification was between ripe syconia of seed and monoecious figs, supporting the idea that they tend to look more similar to each other than to gall syconia (Table 5).

### 3.3 Differentiation of gall and seed syconia with ontogeny (prediction 4)

The first two principal components (PCs) explained 60% of variance in syconia and generally reflected the differences one would expect in the maturation of any fruit (e.g., increases in size and volatile compounds, softening of the pulp, change of pigmentation; Table 6). Confirming the results of our previous analyses between gall and seed syconia at the flower and ripe fruit stages, differentiation between gall and seed syconia appears to occur during the fruits' ontogeny after pollination, especially at ripening; the mean Euclidean distance in a bivariate space defined by the first two PCs between gall and seed syconia of the same species showed an increasing trend from flower, to unripe, to ripe syconia (Table 7A), with most of the differentiation occurring at maturation. The variables that define the third PC do not obviously match traits one would expect to change with fruit maturation. However, because the third PC explains a fairly substantial amount of variance (24%), we included it in a similar analysis based on Euclidean distance in trivariate space. The overall trend is similar to that with the bivariate Euclidean distance, except that one of the species, *F. congesta,* does not follow the expected pattern (Table 7B). Because the fourth PC explained only an additional 10% of the variance, we did not include it in distance calculations.

**Table 6:** Results of PCA to ordinate syconia of different sexes (seed vs. gall syconia) at different developmental stages: flower, immature, and mature fruit

Variables	PC1	PC2	PC3	PC4
Pulp softness	0.406	−0.166	−0.582	0.336
Diameter	−0.376	0.403	−0.399	0.565
Odor	0.220	0.370	−0.510	−0.695
Hue	0.502	−0.448	−0.147	0.164
Saturation	0.520	0.273	0.458	0.160
Brightness	−0.349	−0.631	−0.098	−0.182
Proportion of variance	0.315	0.287	0.236	0.102
Cumulative proportion	0.315	0.603	0.838	0.941

Note

The three species included in this part of the study were *Ficus botryocarpa, Ficus congesta, and Ficus pungens*.

**Table 7:** Euclidean distance between gall and seed figs (*Ficus* sp.) at the different stages of syconium ontogeny in a (A) bivariate space defined by the first two principal components, and (B) trivariate space defined the first three principal components

(A)	Flower	Immature fruit	Mature fruit	(B)	Flower	Immature fruit	Mature fruit
*F. botryocarpa*	0.929	0.925	3.713	*F. botryocarpa*	1.321	1.375	3.713
*F. congesta*	0.872	0.626	0.930	*F. congesta*	0.908	0.987	0.930
*F. pungens*	0.865	1.255	4.240	*F. pungens*	2.136	1.498	4.240
Average	0.889	0.935	2.961	Average	1.203	0.777	3.697

Note

Only the three species for which we had data for all three stages of ontogeny were included. Variables included in the analysis are pulp softness, diameter, total volatile compounds, hue, chroma, and brightness. In two out of three species included gall and seed syconia differentiate well at the mature fruit stage, while at the flower and immature fruit stages they do not differentiate well. Results from *F. congesta* do not show this trend as clearly throughout its development.

### 3.4 Frugivore visits to figs (prediction 5)

Three out of four monoecious trees (75%) were visited by frugivores during video recording, 22 out of 68 seed trees (32%) were visited, and only one out of 20 gall trees (5%) was visited. The one gall tree that was visited had only a single visit by a single disperser, a bandicoot (unidentified to species, family Preamelidae). The bandicoot was not observed consuming a fruit but, following protocol, we recorded its presence as a visit because we could not clearly observe it the entire time in the tree. In support of prediction 5, monoecious and seed trees were visited significantly more often than gall trees (*χ* ^2^ = 9.76, *df* = 1, *p* = .008). A comparison between seed and gall figs indicated that the former received more visits than the latter (*χ* ^2^ = 4.30, *df* = 1, *p* = .038). Visitors consisted of a variety of birds and bats (mostly unidentifiable to species) and very few bandicoots.

Visitation rates to seed figs were 0.021 visits/hr; to gall figs, it was 0.003 visits/hr; and to monoecious figs, it was 0.058 visits/hr. We consider these rates to be rough estimates and conservative because, as mentioned previously, when several frugivores visited the tree in a short period of time, we counted them as one visit even though they could have been by different individuals. Also, it is almost certain that some visits were undetected by our cameras.

## 4 DISCUSSION

Our results support most predictions derived from Lambert's (1992) and Dumont et al.'s (2004) hypothesis about differences and similarities in selective pressures on fig syconia of different types (male vs. female and flower vs. fruit stages). Syconia at the flower stage were similar in both sexes, as expected under selection to attract the same pollinators. We detected no differences in odor, color, and diameter at the flower stage. This “mimicry” between gall and seed figs presumably increases both the female and male components of fitness. Without it, pollen‐loaded female wasps would likely visit gall figs exclusively, as they would be under strong selection to avoid the reproductive dead‐end represented by female, seed syconia. This scenario would not necessarily be beneficial for male figs, as they need the pollen‐loaded female wasps that emerge from them to enter female figs for their gametes to produce viable seeds (Grafen & Godfray, 1991). In any case, the mimicry between seed and gall figs is apparently effective; pollinators visit gall and seed syconia at the flower stage with equal frequency (Dumont et al., 2004; Patel et al., 1995; Weiblen, Yu, & West, 2001).

Syconia at the ripe fruit stage differed significantly between seed and gall figs in traits known to affect frugivore consumption of figs (Lomascolo et al., 2010). Seed syconia tended to show darker and more saturated colors (related to greater amount of pigments), contrasted more against the background, were softer, and emitted more VOCs (related to stronger odor). These results align with previous studies that described differences between seed and gall figs in other traits that often attract seed dispersers, such as syconia nutritional content (Dumont et al., 2004; Weiblen et al., 2010) and diameter (Dumont et al., 2004; Lambert, 1992). Differences in color and odor were also assessed by Dumont et al. (2004), although qualitatively, and likewise found to differ between seed and gall syconia. As predicted, seed syconia generally exhibited traits that should make them more attractive to seed dispersers. Summarizing results from all studies, including ours, seed syconia at the fruit stage are more nutritious, have more pigments, are more odorous, and are softer than gall syconia. The only exception we could find is reported by Dumont et al. (2004), who showed that hardness of syconia did not differ between seed and gall figs. This difference between our results and those of Dumont et al. may be real (i.e., the different species of figs in the two studies have different patterns of fig hardness) or due to differences in methodology. While Dumont et al. (2004) measured hardness as the force needed to puncture the skin, apparently at a small point, we measured it as the overall softness of the entire syconium, which we believe better represents the difference between the rubbery, fibrous pulp of gall syconia and the fleshy, moist pulp of seed syconia. Another difference that we observed (but did not quantify) was the presence of latex in gall syconia, which, together with typically dry, rubbery pulp, might deter frugivores.

Because the traits that distinguish seed syconia are those known to attract seed dispersers of *Ficus*, because those seed dispersers consume seed syconia far more often than gall syconia (this study, Dumont et al., 2004; Chen et al., 2017, but see Phua & Corlett, 1990), and because gut passage of fig seeds is known to enhance their germination (Chen et al., 2017), the differences between seed and gall syconia are consistent with selection by frugivorous seed dispersers. This idea is further supported by our finding that ripe seed syconia tend to have traits that are more similar to those of ripe monoecious fig species, which should also be under selective pressure to attract seed dispersers, than to their conspecific gall figs. We also show that frugivores are much more often attracted to monoecious and seed figs than they are to gall figs, which suggests that differentiation in syconia traits at the fruit stage leads to enhancement of the female function in seed and monoecious figs. It remains to be tested whether consumption by frugivores leads to differences in fitness.

Including odor as a variable that may have evolved differently in gall versus seed figs is important because volatiles are known to influence the behavior of both pollinators and seed dispersers. However, a limitation of our study is that we used a relatively crude measure of volatiles, total amount, not the amounts and identities of individual volatile compounds, many of which are known to be important to mutualists. (Borges et al., 2008; Hossaert‐McKey, Soler, Schatz, & Proffit, 2010; Raguso, 2008). We acknowledge that a thorough test of differentiation between gall and seed syconia at the flower stage would include the component VOCs. Even so, the other variables included in our analyses did not yield significant differences between the fig sexes, whereas they did show differentiation in later stages of fig maturity, and therefore the conclusions that gall and seed figs differentiate with ontogeny holds, even with a relatively simple measure of syconium odor.

Our results also demonstrate, as predicted, that seed dispersers are attracted far more frequently to monoecious and seed figs than to gall figs. Although previous studies suggest a preference of frugivores for seed figs over gall figs (Chen et al., 2017; Dumont et al., 2004; Lambert, 1992), we know of no other study that systematically quantified diurnal and nocturnal visitation to gall and seed fig trees, and that included monoecious figs. With almost 770 hr of observations from a total of 50 trees, we feel confident in concluding that monoecious and seed figs are visited by frugivores far more often than gall figs. Gall figs, however, are sometimes visited (*n* = 1 visit in this study), and gall syconia may be consumed or dispersed at least occasionally (Phua & Corlett, 1990). Three additional lines of evidence support frugivore preference for seed figs over galls figs. First, Dumont et al. (2004) showed that captive bats preferred seed syconia over the gall syconia of one fig species, *F. pungens*. Second, Lambert (1992) reported that during 10 hr of daytime observations of gall figs (the number of trees was not specified) of two species (*Ficus parietalis* and *Ficus obscura*), birds approached the trees but were never seen consuming a syconium. Third, Lambert (1992) counted 1,154 gall syconia of one *F. parietalis* tree and recovered most of them (1,128) as they fell to the ground uneaten. Chen et al. (2017) recorded most bat activity, as measured by pellets and feces with viable seeds, left mostly under female trees of *Ficus septica*; conspecific trees with gall syconia rarely showed the same signs of visitation by bats. We conclude that differentiation of gall and seed syconia at the fruit stage almost certainly enhances the male component of fig fitness, as it greatly reduces the risk of consumption of pollinators in seed figs by seed dispersers. It may simultaneously enhance the female component of fitness, as seed figs are not competing with gall figs for the frugivores that disperse their seeds (Lambert, 1992).

A limitation of our study is that predictions for differences between seed and gall syconia were based on potential selective pressure of seed dispersers in general even though, for example, birds and mammals may exert very different, potentially conflicting selective pressures on fruit traits. We did not differentiate between bird and mammal‐dispersed species because we did not have enough data for each disperser type for all stages of maturity. This renders our analysis conservative and even so, we found that fruit‐stage seed syconia are more similar to each other than to fruit‐stage gall syconia. Moreover, although bats often find and consume figs without the typical VOC profile or the overall characteristics associated with mammal dispersal (Lomascolo et al., 2010), we found that they did not visit gall figs. Thus, it seems reasonable for purposes of this study to have combined all seed figs into a single group attractive to frugivores.

In summary, our results and those from previous studies suggest that figs' response to opposing selection by pollinator and seed disperser mutualists leads to overall similarity of syconia at the flower stage and differentiation at the ripe fruit stage, ensuring pollination of all fig types (Dumont et al., 2004; Lambert, 1992; Lomascolo et al., 2010) and to differential attractiveness to seed dispersers between gall and seed syconia.

In a more general context, our results extend prior work on the evolutionary advantages of dioecy. Dioecy is thought to be facilitated by pollinator‐mediated selection for floral dimorphism and driven by avoidance of selfing and optimization of resource allocation to male and female structures (Ashman, 2000; Charlesworth, 1999; Freeman, Doust, El‐Keblawy, Miglia, & McArthur, 1997). Worldwide, dioecy is associated with climbing growth form, abiotic pollination, and animal‐mediated seed dispersal (Renner & Ricklefs, 1995). These associations do not generally occur in our sample of *Ficus* species in Papua New Guinea, a region of especially high *Ficus* diversity. Although some of our species start out as climbers, they afterward become free‐standing trees (most species strangle their host), and all of them are insect‐pollinated. Consistent with worldwide patterns, however, all are animal‐dispersed. In *Ficus,* the exceptions to traits generally associated with dioecy are almost certainly related to its unusual life history. In particular, effective breeding populations are extraordinarily large (i.e., gene flow is high and selfing presumably low) and males produce fruit‐like structures, which uniquely require allocation of parental resources (Herre, Jander, & Machado, 2008).

Mutualisms may be defined as antagonisms in equilibrium, as both partners exploit the other to maximize their own benefits; tight interactions evolve only if benefits exceed costs for both partners (Bronstein, 2015; Morris, Vazquez, & Chacoff, 2010). Figs provide an excellent example. Their pollinators consume seeds that the pollinators, themselves, helped create. Some plants “pay” for pollination services with nothing more than a deadly trap, and their seed dispersers may eat the pollinators inside the fruits. Likewise, some species of fig wasps are floral parasites, depending on fig seeds but not pollinating fig flowers (Duthie, Abbott, & Nason, 2015). Further studying the mechanisms through which figs strike an evolutionary balance between avoiding detrimental interactions with fig wasps and frugivores while obtaining the benefits of pollination and seed dispersal will help reveal how equilibria in mutual exploitation are achieved, both in specialized interactions (figs and their pollinators) and more generalized ones (figs and seed dispersers).

## CONFLICT OF INTEREST

None declared.

## AUTHOR CONTRIBUTIONS

SBL conceived the idea, designed, and performed fieldwork and statistical analyses. DJL helped to shape the idea, design the methods and fieldwork, and frame the study in theory. SBL and DJL wrote the manuscript.

## Data Availability

All data needed to repeat the analyses in this paper are presented in Tables 1, 2, 3. Additional data will be freely available upon request.
